# High-Porosity Hydrogel Microneedles for Rapid and Efficient Extraction of Imidacloprid Residues in Peach Fruits

**DOI:** 10.3390/foods14193423

**Published:** 2025-10-04

**Authors:** Chi Zhang, Xin Liu, Tailong Cai, Jianfeng Ping, Yibin Ying

**Affiliations:** 1 Laboratory of Agricultural Information Intelligent Sensing, College of Biosystems Engineering and Food Science, Zhejiang University, Hangzhou 310058, China; czhanggeorge@zju.edu.cn (C.Z.); liuxin8234@zju.edu.cn (X.L.); l210625@zju.edu.cn (T.C.); jfping@zju.edu.cn (J.P.); 2 ZJU-Hangzhou Global Scientific and Technological Innovation Center, Zhejiang University, Hangzhou 311215, China; 3 Zhejiang Key Laboratory of Intelligent Sensing and Robotics for Agriculture, Hangzhou 310058, China

**Keywords:** high-porosity, hydrogel microneedles, extraction, pesticide, peach

## Abstract

Accurate and rapid extraction of pesticide residues in fruits is crucial for timely food safety monitoring. However, conventional extraction methods remain labor-intensive and time-consuming, often requiring hours for sample preparation. Here, we present a porous hydrogel microneedle (HMN) patch integrated with an automated insertion applicator as a highly efficient platform for the rapid extraction of peach juice for imidacloprid residue detection. The HMN patch, composed of polymethyl vinyl ether/maleic anhydride (PMVE/MA) polymer, was fabricated with high porosity by adjusting the porogen content. Under optimized porogen content of 3% NaHCO_3_, the developed HMN patch exhibited ultrahigh extraction efficiency, achieving a 40-fold water absorption capacity and extracting 0.6% (*w*/*w*) peach solids of its weight within 5 min. The extracted juice could be readily recovered through a simple elution process and was directly compatible with both high-performance liquid chromatography (HPLC) analysis and lateral flow assays. Compared with conventional destructive methods, the HMN platform offers a scalable, high-efficiency, and user-friendly solution for high-throughput pesticide extraction. The integration of the automated applicator further enhances consistency, minimizes user variability, and facilitates on-site monitoring of pesticide residues, providing a practical pathway for field-deployable food safety monitoring.

## 1. Introduction

Pesticide residues in fresh produce have emerged as a global food safety concern due to their potential risks to human health and the environment [1], as well as their impact on international trade and regulatory compliance [2,3,4]. Among these pesticides, neonicotinoids such as imidacloprid are widely applied in horticulture owing to their systemic action and broad-spectrum insecticidal properties [5,6]. However, their persistence in fruits like peaches frequently leads to detectable residue levels, sometimes exceeding maximum residue limits established by agencies [7,8]. Although this kind of pesticide has low toxicity, its ingestion can interfere with the human nervous system, and long-term exposure may lead to severe chronic toxicity [9]. For imidacloprid in peaches, China’s national standard GB-2763 [10] sets limits for pesticide residues of 0.5 ppm. The rising stringency of global regulatory frameworks, combined with growing consumer awareness, underscores the urgent demand for rapid, reliable, and scalable methods to monitor pesticide residues in fruit supply chains [11,12].

Traditional pesticide detection workflows predominantly involve destructive sampling and solvent-based extraction followed by chromatographic techniques such as high-performance liquid chromatography (HPLC) or gas chromatography coupled with mass spectrometry (GC-MS) [13,14]. These methods are well-established and highly sensitive, yet they are time-consuming and labor-intensive, often requiring multiple preprocessing steps including homogenization, centrifugation, and solvent partitioning, which can take several hours per batch [15]. Moreover, these protocols demand specialized equipment and trained personnel, limiting their applicability for on-site monitoring and high-throughput screening in agricultural or distribution settings. Consequently, despite their analytical robustness, conventional methods fail to meet the increasing need for rapid and field-deployable residue analysis.

Recent advances have sought to overcome these limitations by developing miniaturized and minimally invasive sampling technologies that simplify sample preparation while maintaining compatibility with established analytical methods [16,17,18]. Among these, microneedle (MN)-based extraction systems have garnered attention for biological and food sampling [19,20]. Originally designed for transdermal drug delivery, MNs have since been adapted for plant and food applications due to their ability to penetrate protective barriers such as fruit cuticles and access intracellular fluids without significant tissue disruption [21,22,23]. By bypassing extensive preprocessing steps, MN-based approaches hold promise for accelerating residue monitoring workflows.

In this work, hydrogel-based microneedles (HMNs) represent a particularly promising class of extraction devices. Hydrogels, composed of crosslinked polymer networks, exhibit intrinsic hydrophilicity, biocompatibility, and capillary-driven fluid absorption, making them well-suited for extracting aqueous matrices like plant sap or fruit juice [24]. Moreover, by engineering highly porous structures, HMNs can dramatically enhance their absorption kinetics and capacity, enabling rapid uptake of analytes [1]. Recent studies have demonstrated the utility of HMNs in fields ranging from biomedical diagnostics to agricultural metabolite monitoring [25,26]. However, most existing systems exhibit limited extraction throughput, slow absorption rates, or inconsistent performance due to manual deployment, restricting their practical utility for large-scale residue detection.

To address these challenges, we reported the development of a porous hydrogel microneedle (HMN) patch composed of polymethyl vinyl ether/maleic anhydride (PMVE/MA) polymer, in which porosity was precisely tuned by controlling porogen content to maximize fluid absorption efficiency. The engineered HMN patch demonstrated exceptional extraction performance, achieving a 40-fold water uptake within seconds and recovering peach juice containing up to 0.6% (*w*/*w*) solids of its own weight within just 5 min. Furthermore, to eliminate operator-dependent variability and ensure reproducible insertion into fruit tissues, the HMN patch was integrated with an automated insertion applicator. This applicator standardized microneedle deployment depth and speed, significantly enhancing sampling consistency and throughput, while reducing reliance on trained personnel.

**Figure 1:**
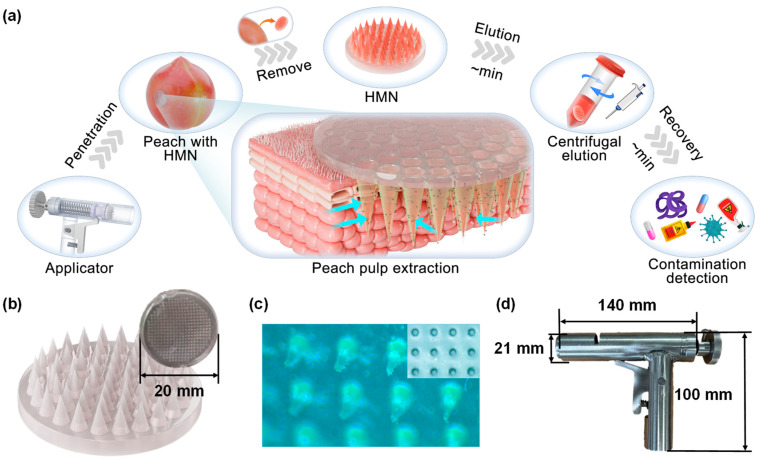
Application of the HMN patch-based rapid extraction system in the extraction of peach pulp juice collection. (**a**) Schematic illustration of the extraction process using the porous HMN patch. (**b**) Macroscopic view and dimensional analysis of HMN patch. (**c**) Array morphology of the HMN observed via CCD imaging. (**d**) Physical view and dimensional characterization of the automated insertion applicator for HMN patch.

The extracted juice could be directly recovered through a simple elution process and was immediately compatible with both HPLC and lateral flow assays, enabling flexible downstream detection of imidacloprid residues (Figure 1a). Compared with conventional destructive extraction techniques, this integrated HMN platform delivers a scalable and time-efficient solution for pesticide residue extraction, thereby facilitating on-site, real-time monitoring of fruit safety and quality. By leveraging the synergistic advantages of high-porosity hydrogel matrices and automated applicator-assisted deployment, this work establishes a versatile and practical pathway for advancing residue detection technologies, with potential applications across fresh produce monitoring and regulatory enforcement.

## 2. Experimental Section

### 2.1. Materials and Reagents

Seasonal peaches were supplied by Shouguang Agricultural Holdings Group Co., Ltd. (Shouguang, China). Polydimethylsiloxane molds were custom-made by Taizhou Microchip Medical Technology Co., Ltd. (Taizhou, China). Poly (methyl vinyl ether-maleic anhydride copolymer) (PMVE-MA, Mw 80,000), polyethylene glycol (PEG), imidacloprid (C_9_H_10_ClN_5_O_2_), and sodium bicarbonate (NaHCO_3_) were purchased from Shanghai Aladdin Biochemical Technology Co., Ltd. (Shanghai, China). Agarose gel and Rhodamine B (RhoB) were purchased from Shanghai Macklin Biochemical Technology Co., Ltd. (Shanghai, China). Anhydrous ethanol and Cell Counting Kit-8 (CCK-8) were respectively provided by National Pharmaceutical Group Chemical Reagents Co., Ltd. (Shanghai, China) and Shanghai Beyotime Biochemical Technology Co., Ltd. (Shanghai, China). These reagents were of analytical grade unless otherwise specified.

### 2.2. Preparation of HMN

A solution was prepared by dissolving 7.5 g of PMVE-MA powder in 75 mL of deionized water at 80 °C under continuous stirring for 12 h. After the solution was cooled to room temperature, 7.5 g of PEG powder and a specified quantity of NaHCO_3_ (≤5%) were added to form the microneedle precursor solution via continuous stirring for 12 h. Then this microneedle precursor solution was cast into a PDMS mold and placed under vacuum (0.10 MPa) for 30 min to eliminate entrapped air. The solution was thermally cross-linked in an oven at 80 °C for 6 h to form a solidified microneedle patch (HMN). As shown in Figure 1b,c, the semi-transparent HMN with a diameter of 20 mm is uniformly covered with complete conical needle tips. All fabricated HMNs were stored in a desiccator. All fabricated HMNs were stored in a desiccator.

### 2.3. Characterization

#### 2.3.1. In Vitro Extraction Property of HMN in a Simulated Tissue Matrix

A simulated tissue matrix was prepared by dissolving 1.4 g of agarose and 0.01 g of RhoB in 98.6 g of deionized water. The mixture was heated to 80 °C in a water bath until it was completely dissolved, then cooled to room temperature and allowed to solidify. The initial dry mass of HMN was recorded. Individual HMN was inserted into the solidified agarose gel and incubated for specified durations (≤180 s) at room temperature.

Following incubation, the HMN was immersed in a known volume (*V*) of deionized water and centrifuged at 7800× *g* for 2 min. A 100 μL aliquot of the supernatant was collected. The recovery efficiency (*E*) was calculated as:

$$E=\frac{C_{d}\times V}{C_{0}\times \Delta W/\rho }\times 100\%$$

where *C_d_* = RhoB concentration in supernatant (μg/mL, measured by absorbance at 552 nm). *V* = Volume of deionized water added for extraction (mL). *C*_0_ = Initial RhoB concentration in the agarose gel (100 μg/mL). Δ*W* = Mass change of the HMN after insertion into the gel and before extraction. *E* = RhoB recovery efficiency (%).

#### 2.3.2. In Vitro Extraction Property of HMN in the Crushed Peach Flesh

Fresh peach flesh was dissected and homogenized to yield 5 g of tissue homogenate. A 5 mL aliquot of imidacloprid standard solution was introduced and thoroughly mixed with the homogenate. The mixture was further processed using a homogenizer for 1 min, followed by centrifugation at 7800× *g* for 2 min at room temperature. HMNs were then fully immersed in the resulting supernatant and incubated for 3 min at room temperature. After incubation, patches were carefully removed. The method for extracting imidacloprid from the HMNs and the method for calculating the recovery efficiency of imidacloprid are consistent with those described in the previous text.

#### 2.3.3. In Vivo Penetration HMN Elution for HPLC

The solution inside the HMN was dissolved in 5 mL of 25% acetonitrile. After centrifugation at 7800× *g* for 2 min and incubating for 3 min, the solution was extracted and supplemented with pure acetonitrile to a final volume of 5 mL. The solution was then subjected to rotary evaporation until nearly dry. The remaining solution was dissolved in 2 mL of 25% acetonitrile for HPLC analysis.

#### 2.3.4. Cell Activity Detection

The punctured parts of peaches were fully ground to extract fruit solids. In total, 0.2 g solids was extracted with 200 μL deionized water, which was put into a centrifuge and rotated for 3 min at the speed of 1200 r/min. Then, 100 μL supernatant was extracted and added to the enzyme label plate together with 10 μL CCK-8. The plate was placed in a constant temperature incubator at 37 °C for 3 h. Then the label plate was placed in the label meter and the absorbance of 450 nm was measured. Cell activity, marked as *CA*, was calculated according to the formula:

$$CA=\frac{C_{c}-C_{B}}{C_{CK}-C_{B}}\times 100\%$$

where *C_C_* is the absorbance of the testing sample, *C_B_* is the one of the blank samples and *C_CK_* is the one of the control cells.

#### 2.3.5. Applicator Fabrication, Assembly and Usage

The automated insertion applicator for HMN patch was designed using *SolidWorks 2018*, and then each component was fabricated using metal manufacturing technology, followed by assembly as shown in Figure 1d. The device primarily consisted of a sleeve, a propulsion rod, a propulsion port, a spring, a limit knob, and a launcher. The propulsion port secured the HMN patch, ensuring it could smoothly detach from the device during launch. The sleeve, propulsion rod, and spring together formed the HMN patch launch power system, responsible for propelling the HMN toward the target tissue with a defined momentum. The limit knob controlled the propulsion stroke, effectively preventing excessive force from being applied to the HMN, thereby reducing the risk of mechanical compression and damage to the peach tissue. This applicator design enabled precise, low-damage insertion of HMNs, facilitating efficient juice extraction for subsequent rapid detection.

#### 2.3.6. Large Instruments Used in the Experiment

The microscopic morphology of HMNs was characterized using scanning electron microscope (SEM, G300, Zeiss, Oberkochen, Germany). Their mechanical properties were tested with a texture analyzer (Stable Micro Systems PLUS, Stable Micro Systems, Godalming, UK) equipped with a P-5 probe. The recovery solutions were analyzed by high-performance liquid chromatography (HPLC, Agilent HPLC 1260, Agilent Technologies, Santa Clara, CA, USA). The soluble solid content of the peaches was measured using a digital refractometer (HP-TD1, Anying, Shenzhen, China). The cellular activity of the peaches was evaluated after staining with a reagent kit, using an enzyme-linked immunosorbent assay reader (ELISA, EX1301, BioTek, Memphis, TN, USA).

## 3. Results and Discussion

### 3.1. Preparation and Structure Characterization of HMN Patch

**Figure 2:**
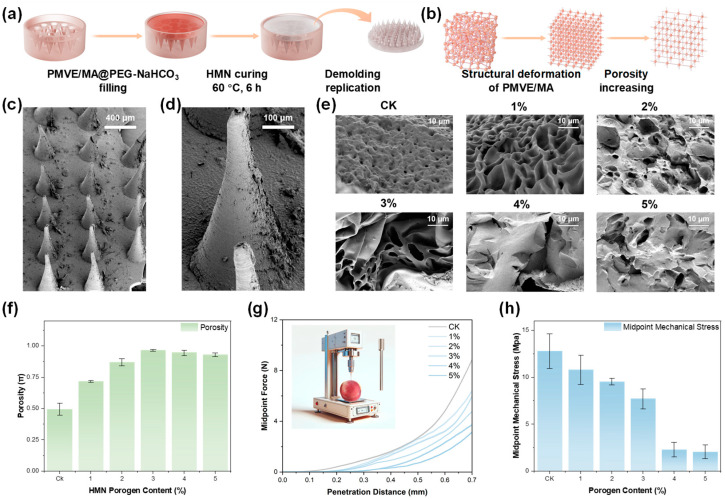
Preparation and structure characterization of HMN patch. (**a**) Schematic illustration of HMN patch fabrication process and (**b**) its variation in the microstructure. (**c**) SEM characterization of HMN arrays and (**d**) single array. (**e**) Morphological structure, (**f**) porosity, and (**g**) mechanical properties of HMN patch with various NaHCO_3_ contents from 0% (CK), 1 wt%, 2 wt%, 3 wt%, 4 wt%, and 5 wt%. (**h**) Comparison of the midpoint mechanical force in the HMN patch with various NaHCO_3_ contents.

The HMN patch was fabricated using a demolding technique, where the precursor solution was cast into a PDMS microneedle mold and cured to form a complete microneedle array (Figure 2a). The role of the NaHCO_3_ porogen in tuning the internal architecture was illustrated in Figure 2b. In the absence of NaHCO_3_, the binding sites between PMVE/MA and PEG transitioned from a disordered to an ordered state upon curing. When NaHCO_3_ was introduced, partial disruption of these binding networks occurred, increasing the interstitial space and thereby enhancing porosity and fluid diffusivity. The macroscopic morphology of the HMN patch was characterized by scanning electron microscopy (SEM). As shown in Figure 2c,d, the MN arrays retained their shape and integrity with or without NaHCO_3_ addition, indicating that the porogen did not compromise mold fidelity. However, SEM images of the internal microstructure (Figure 2e) revealed that NaHCO_3_ significantly altered the porous morphology. The MN arrays in the control group (CK) fabricated without NaHCO_3_ porogen exhibited only a dense structure with small pores. Upon addition of 1 wt% porogen, the interior of the MN developed an interconnected porous structure with significantly increased diameter. As the porogen concentration further increased, the pore diameter progressively expanded until structural collapse occurred. Notably, a well-defined and stable porous architecture was observed only at 3 wt% porogen content, suggesting that this specific content facilitated the formation of optimally large yet structurally stable pores.

Porosity changes were further validated by density measurements (Figure 2f). Porosity measurements demonstrated that the baseline HMN arrays (0 wt% NaHCO_3_) possessed about 50% porosity. Systematic porogen incorporation led to a content-dependent porosity enhancement, achieving peak porosity (97%) at the optimal 3 wt% NaHCO_3_ content, reaching the optimal levels that theoretically maximize extraction efficiency while maintaining structural integrity. Subsequent porogen increases beyond this threshold showed negligible effects on porosity, consistent with the pore structure characteristics revealed by SEM analysis. However, this porosity enhancement came at the cost of mechanical performance. Texture analysis demonstrated decreasing compression modulus with higher porogen contents (Figure 2g). To further assess mechanical performance, halfway mechanical force tests were conducted to reveal critical performance thresholds (Figure 2h). When NaHCO_3_ content reached 4 wt%, the halfway force dropped to nearly one-third of that observed in the 3 wt% group, indicating compromised structural stability. The compromised mechanical properties significantly reduced the demolding success rate for the MN patch fabricated above this critical threshold, as the increased material brittleness and reduced toughness promoted structural fractures during the release process. These results established a clear trade-off between extraction-enhancing porosity and mechanical reliability. The optimal NaHCO_3_ content (3 wt%) balanced substantial porosity with acceptable mechanical strength, while higher NaHCO_3_ content risked structural failure during both manufacturing and application.

### 3.2. Swelling and Extraction Properties of HMN Patch

**Figure 3:**
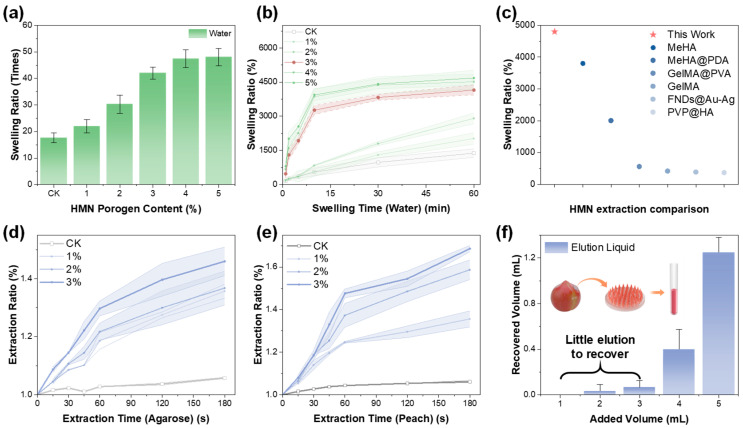
Swelling and extraction properties of HMN patch. (**a**) Maximum swelling capacity (within 1 day) and (**b**) optimized swelling time (within 60 min) of HMN with various NaHCO_3_ contents from 0% (CK), 1 wt%, 2 wt%, 3 wt%, 4 wt%, and 5 wt%. (**c**) Comparison of swell performance between other reported HMN and this work. HMN extraction ratio of (**d**) agarose gel and (**e**) contaminated peach juice within 180 s. (**f**) Quantitative analysis of eluent volume versus recovery yield during the 5 min extraction process.

The swelling behavior of the HMN patch was systematically evaluated to assess its extraction potential. HMN patches with various NaHCO_3_ porogen content were immersed in water for 24 h to ensure full hydration, and their mass ratios before and after swelling were measured (Figure 3a). Hydration studies revealed that the HMN patch without porogen exhibited a 15-fold mass increase after 24 h of water immersion, while NaHCO_3_-modified HMN patches showed markedly higher swelling capacities with the increasing NaHCO_3_ content and reached a plateau at swelling capacity (40-fold mass increase) at 3 wt% porogen content. These results demonstrated the critical role of porogen incorporation in enhancing fluid absorption capacity. For practical fruit extraction applications, short-term swelling kinetics proved particularly important. Figure 3b showed the swelling ratio (*S*) of each formulation during the first hour, calculated as:

$$S=\frac{W_{t}}{W_{0}}\times 100\%$$

where *W*_0_ and *W_t_* were the initial dry mass and the swollen mass at time *t*, respectively. All prepared HMN patches exhibited a progressive increase in *S* over time, with higher porogen content leading to faster swelling and earlier saturation of maximum capacity, while the swelling ratio showed only a slight increase when the porogen content increased from 3 wt% to 5 wt%, confirming the establishment of a performance plateau as previously demonstrated. Comparative analysis with recently reported HMN systems highlighted the superior performance of our HMN prepared with PMVE/MA@PEG and NaHCO_3_ [27,28,29,30,31], suggesting its strong potential for fruit fluid extraction.

The solid-phase extraction capacity was next assessed using agarose gel as a model (Figure 3d). HMN patch was inserted for 3 min and weight gain was recorded. Consistent with swelling behavior, higher NaHCO_3_ content correlated with greater gel uptake. Practical validation using fresh peaches (3 min insertion, Figure 3e) reproduced these trends, with porogen-modified HMN patch consistently outperforming conventional HMN. The HMN patch with optimized 3 wt% NaHCO_3_ formulation demonstrated superior efficiency in fluid uptake capacity, extracting more than 0.6% (*w*/*w*) peach solids. Post-extraction, HMN required elution to recover the target analyte. Therefore, the elution process was carefully optimized for imidacloprid recovery. The QuEChERS method was adopted as the standard extraction and recovery approach of excised fruit tissues, utilizing acetonitrile as the extraction solvent. To validate the HMN-based extraction, the eluent used to recover imidacloprid from HMN was prepared using 25% acetonitrile for subsequent analysis, following the QuEChERS protocol. Using the optimized HMN patch and 25% acetonitrile as the eluent, a rapid elution procedure was employed. After sample collection, the HMN patch was fully immersed in the eluent, subjected to oscillating at 7800× *g* for 2 min to facilitate analyte release, and subsequently left standing for 3 min to allow complete diffusion and solvent penetration before recovery. The effect of eluent volume on recoverable fluid was examined. As shown in Figure 3f, volumes below 3 mL yielded insufficient recovery for analysis, whereas 5 mL enabled ~1.2 mL recovery that was adequate for downstream detection (Figure 3f). Under this condition, the RSD value of the recoverable solution volume was 10% based on 5 replicates. This extraction method provides a sufficient volume of peach juice for subsequent analysis; therefore, a 5 mL eluent volume was adopted for the following HMN recovery. The optimized protocol ensured both speed and efficiency in analyte recovery, supporting the practical applicability of HMN in rapid fruit fluid sampling. The entire extraction-elution workflow was successfully completed in 5 min, representing a significant improvement over conventional methods requiring hours of processing.

### 3.3. Practical Extraction Performance of HMN Patch for Peach Juice Detection

**Figure 4:**
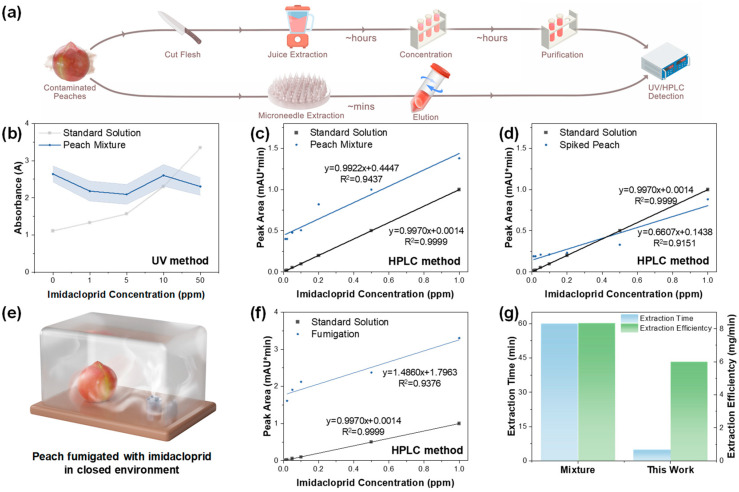
Practical extraction performance of the HMN patch for imidacloprid detection. (**a**) Comparison of the workflow and time required to extract peach juice from contaminated peaches using the traditional extraction method and the HMN patch extraction method. (**b**) UV-vis spectral and (**c**) HPLC analysis comparison between imidacloprid standard solution and peach juice spiked with imidacloprid standards. The samples were obtained by the HMN patch extraction method. (**d**) HPLC analysis of HMN-extracted peach juice from imidacloprid-spiked peach (injection-spiking method). (**e**) Schematic illustration of peach fumigation with imidacloprid in a sealed chamber. (**f**) HPLC validation of HMN-extracted peach juice from imidacloprid-spiked peach (fumigation-spiking method). (**g**) Comparison of extraction time and efficiency between the traditional sampling method and the fumigation-spiking method.

The HMN patch enables rapid and efficient extraction of peach juice compared with conventional approaches. As shown in Figure 4a, traditional approaches required multiple processing steps including fresh cutting, homogenization, concentration, and purification, typically consuming several hours per sample. In contrast, the HMN-based extraction method achieved comparable analyte recovery within 5 min through a simple insertion and recovery process, while preserving fruit integrity.

To further assess its analytical applicability, the performance of the HMN patch was evaluated for the detection of imidacloprid residues in extracted peach juice. Initial screening by UV-Vis spectrophotometry demonstrated a positive correlation for imidacloprid standard solutions (Figure 4b). However, significant signal drifting was observed in peach juice samples spiked with imidacloprid standards, which may be caused by matrix interference in the peach juice. This suggested that UV-vis spectrophotometry may be unsuitable for practical residue analysis in complex fruit matrices. To establish a reliable detection protocol, we employed HPLC analysis following two complementary validation approaches. First, controlled spiking studies were conducted using either direct addition to peach juice (Figure 4c) or tissue injection (Figure 4d), followed by peach juice extraction using an HMN patch and analysis by HPLC. Both methods yielded excellent agreement with theoretical concentrations, confirming the method’s accuracy under controlled conditions.

To evaluate environmental relevance, a custom fumigation system was developed to simulate field-acquired contamination while preserving fruit integrity. Specifically, peaches were placed in a sealed chamber and exposed to vaporized imidacloprid generated by a fumigation device (Figure 4e), followed by HMN patch extraction and HPLC analysis. The calibration curve exhibited distinctly different increasing trends at low (0.02–0.1) and high (0.1–1.0) analyte concentrations. The trend observed at higher concentrations closely resembled that of the calibration curve obtained from the standard solution (*R*^2^ = 0.9999). This phenomenon was attributed to the matrix effect of peach juice, which exerted a stronger influence on low-concentration analytes than on high-concentration ones, thereby leading to this segmented response. Nevertheless, within the analyte range of 0.02–1.0 ppm, the overall HPLC response still maintained a linear relationship (*R*^2^ = 0.93) (Figure 4f), demonstrating robust analytical performance against complex matrices. According to the detection results shown in the figure, the relationship between the HPLC absorbance peak area and the imidacloprid concentration in peach was expressed as y = 1.4860x + 1.7963. From the results, it can be concluded that the microneedle extraction method for peach juice achieves a Limit of Detection (LOD) of 0.019 ppm and a Limit of Quantitation (LOQ) of 0.064 ppm, when analyzed by HPLC. The above analysis confirmed that HMN could efficiently extract and enable reliable detection of pesticide residues under practical environmental exposure conditions. For comparison, we also conducted the traditional peach juice extraction method to prepare samples to be analyzed. Finally, Figure 4g shows a comparison between the traditional method and the HMN-based extraction method in terms of extraction time and efficiency. The HMN extraction protocol reduced processing time from several hours to 5 min while maintaining considerable extraction efficiency, establishing its practical superiority for rapid field monitoring. This comprehensive validation confirmed the HMN system’s reliability for both controlled and environmentally acquired imidacloprid residues in fresh produce.

### 3.4. Design of HMN Patch Insertion Applicator and Rapid Detection System

When extracting peach juice with HMN patch, the needles must pierce the fruit surface to access the juice. Conventional operation requires pressing the HMN patch against the peach, inevitably causing obvious compression damage resulting from the deformation of the soft peach tissue under pressure. Such damage compromises tissue integrity, increases the risk of microbial infection, and accelerates fruit spoilage, thereby limiting the practical application of HMN in fruit sampling.

**Figure 5:**
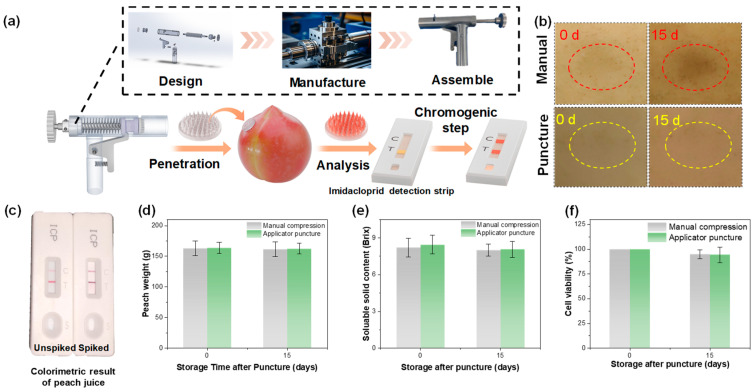
Design of HMN patch insertion applicator and rapid detection system. (**a**) Schematic illustration of the HMN patch insertion applicator design process and the integrated rapid detection system combining HMN-based fluid extraction with lateral flow strip detection. (**b**) Wound changes after 15 days of storage following the HMN patch puncture into the peach epidermis using manual compression and applicator assistance. (**c**) Results of lateral flow strip detection for the national standard concentration of unspiked and spiked peach juice sample extracted using HMN patch. (**d**) Peach weight changes, (**e**) local SSC, and (**f**) local cell viability of peaches after 15 days of storage following epidermal puncture with the HMN patch using either manual compression or applicator puncture.

To overcome this limitation, we developed a high-impulse propulsion HMN insertion applicator that used the HMN’s momentum to puncture the peach epidermis, achieving effective penetration while avoiding compressive forces, as shown in Figure 5a. By integrating this applicator-based HMN extraction method with a lateral flow strip-based detection module, we established a complete low-damage rapid detection system. After the HMN patch extracted peach juice, centrifugal elution was used to recover the sample, which was then applied directly to the competitive lateral flow strip for enabling semi-quantitative identification of imidacloprid levels. The analyte in the sample and immobilized artificial antigens on the test line (T) compete for binding to limited antibody–gold conjugates, resulting in an inversely proportional signal intensity to analyte concentration. A control line (C) confirms valid assay execution by capturing unbound conjugates.

The practicality of this low-damage rapid detection system was verified by comparing peach appearance after puncturing with either the manual compression or the applicator. As illustrated in Figure 5b, compression puncturing caused surface indentations that developed browning after 15 days of storage, whereas applicator puncturing left no visible changes over the same period. Colorimetric detection further confirmed the system’s efficacy. As depicted in Figure 5c, juice from fumigated peaches produced a clear positive signal, while juice from untreated peaches yielded a negative result. To evaluate the impact on fruit quality, weight change, and internal physiological indices were measured. Figure 5d shows that weight loss in applicator-punctured peaches was minimal and comparable to that of the manual compression method. Soluble solid content (SSC) at puncture sites (Figure 5e) remained close to control levels, with negligible changes during storage. Cell viability analysis (Figure 5f) revealed that applicator puncturing preserved metabolic activity, whereas compression puncturing caused significant local browning and slight viability loss.

Overall, the applicator served to ensure consistent and efficient penetration of the HMN patch into the peach skin, reducing manual variability and minimizing potential damage to surrounding tissues. By enabling precise and reproducible patch deployment, the applicator enhanced the stability of sample extraction conditions, thereby improving the reliability of downstream analysis. This design is integrated with the rapid detection system, as it facilitates uniform sample acquisition and shortens preparation time, ultimately contributing to higher throughput and accuracy in biochemical assessments. This controlled deployment preserves sample integrity by eliminating the collateral damage associated with manual application. The applicator’s rapid operation (<10 s per sample) synergizes with the test strip’s 15-min analysis window, establishing a workflow from minimally invasive sampling to actionable results. This combined system addresses the key challenges of field-deployable pesticide monitoring by maintaining analytical reliability while overcoming the traditional trade-offs between detection accuracy and produce preservation.

## 4. Conclusions

In this study, we developed HMNs using a PMVE-MA-based polymer matrix integrated with NaHCO_3_ as a porogen. Through systematic optimization, 3 wt% NaHCO_3_ yielded HMNs with ultrahigh porosity (~97%), significantly enhancing fluid uptake capacity. The resulting HMNs achieved a 40-fold water absorption within seconds and extracted 0.6% (*w*/*w*) peach solids within 5 min, demonstrating excellent extraction efficiency. The extracted juice was efficiently recovered via rapid elution and analyzed using both HPLC and lateral flow assays, validating the compatibility of HMN-extracted samples with standard or rapid imidacloprid detection methodologies. Furthermore, to enable rapid, precise, and low-damage sampling, we engineered an automated high-impact applicator that standardized insertion depth and minimized fruit tissue compression. When coupled with commercial colorimetric strips, this integrated system achieved on-site, semi-quantitative detection of imidacloprid residues within 15 min. Collectively, this HMN-based platform offers a scalable and minimally invasive solution for high-throughput pesticide extraction.

## Data Availability

The original contributions presented in this study are included in the article. Further inquiries can be directed to the corresponding author.
